# Anionic Long-Circulating Quantum Dots for Long-Term Intravital Vascular Imaging

**DOI:** 10.3390/pharmaceutics10040244

**Published:** 2018-11-20

**Authors:** Haolu Wang, Haotian Yang, Zhi Ping Xu, Xin Liu, Michael S. Roberts, Xiaowen Liang

**Affiliations:** 1 Therapeutics Research Group, The University of Queensland Diamantina Institute, The University of Queensland, Translational Research Institute, Brisbane, QLD 4102, Australia; h.wang21@uq.edu.au (H.W.); haotian.yang@uq.net.au (H.Y.); xin.liu@uq.edu.au (X.L.); m.roberts@uq.edu.au (M.S.R.); 2 Australian Institute for Bioengineering and Nanotechnology, The University of Queensland, St Lucia, Brisbane, QLD 4072, Australia; gordonxu@uq.edu.au; 3 School of Pharmacy and Medical Science, University of South Australia, Adelaide, SA 5001, Australia

**Keywords:** quantum dots, blood vessels, intravital imaging

## Abstract

A major impediment to the long-term in vivo vascular imaging is a lack of suitable probes and contrast agents. Our developed mercaptosuccinic acid (MSA) capped cadmium telluride/cadmium sulfide (CdTe/CdS) ultrasmall quantum dots (QDs) have high fluorescent quantum yield, long fluorescence lifetime and long half-life in blood, allowing high resolution long-term intravital vascular imaging. In this study, we showed that these QDs can be used to visualize the in vivo the vasculature in normal and cancerous livers in mice using multiphoton microscopy (MPM) coupled with fluorescence lifetime imaging (FLIM), with cellular resolution (~1 µm) up to 36 h after intravenous injection. Compared to highly regulated and controlled sinusoids in normal liver tissue, disordered, tortuous, and immature neovessels were observed in tumors. The utilized imaging methods have great potential as emerging tools in diagnosis and monitoring of treatment response in cancer.

## 1. Introduction

Optical-based intravital imaging of vasculature is an emerging modality for studying vascular structure, function, and angiogenesis. Changes in the number and spacing of vessels, permeability of the vasculature and vascular function have been implicated in many diseases, including malformation and cancer [1]. Although many probes and contrast agents have been developed for imaging of microvasculature, issues related to stability and bioavailability have yet to be overcome [1,2]. A multivalent and biologically compatible platform for the development of fluorescent imaging agents is still needed for long-term in vivo imaging. As a biological imaging agent, quantum dots (QDs) possess a number of distinct advantages over other nanoparticles (NPs) including high quantum yield, enhanced photostability, narrow emission band, and long fluorescence lifetime [3,4,5]. QDs applied for in vivo imaging of tumor vasculature were firstly reported by Cai et al. [6], where the majority of QDs were found in the tumor vasculature with few binding to tumor cells. Smith et al. also reported the binding events of QDs to the tumor vasculature using intravital microscopy [7]. This study confirmed that QDs did not extravasate but only targeted the vascular integrin αVβ3, which is involved in tumor angiogenesis. However, these studies only reported in vivo QDs distribution in tumors at the organ-level or by ex vivo sections. The limited resolution and penetration depth of conventional in vivo imaging techniques make it difficult to obtain a clear real-time dynamics of QDs at the cellular level in vivo.

We have successfully synthesized long-circulating QDs in our laboratory and their in vivo fate has been fully investigated [4,5,8]. Our results showed these QDs mainly distributed in the liver and kidney. We then further investigated the spatiotemporal disposition of QDs in the liver and kidney by multiphoton microscopy (MPM) [4]. These QDs were evenly distributed in the blood vessels of these organs and circulated for a long time, but were not taken up by hepatocytes and tubular cells [4]. Here, we further applied these ultrasmall water-dispersible cadmium telluride/cadmium sulfide (CdTe/CdS) QDs for the long-term intravital imaging of vasculature in normal and cancerous livers.

## 2. Materials and Methods

### 2.1. Chemicals and Cells

Ilium xylazile and ketamine hydrochloride were purchased from Bayer Australia (Pymble NSW, Australia). Hepa 1-6 cells were obtained from ATCC (Manassas, VA, USA) and maintained in vitro under cell culture conditions recommended by ATCC.

QDs were mercaptosuccinic acid (MSA, Sigma-Aldrich, MO, USA) capped cadmium telluride/cadmium sulfide (CdTe/CdS, Sigma-Aldrich) particles, recently developed in our laboratory [4]. These QDs have an average particle diameter of 3.5 nm determined by transmission electron microscopy (JEOL1010, JEOL, Tokyo, Japan). The hydrodynamic diameter of these QDs was measured to be 4.2 nm. They display abroad excitation (<500 nm) with an emission peak of about 630 nm. The zeta potential measured was −37 mV [4].

### 2.2. Animal Models

All animal procedures were approved by the health sciences ethics committee of the University of Queensland (ethics number is 521/12 and approved on the year of 2013) and were carried out in accordance with the legislation of Australian authorities for the care and use of experimental animals.

Male 8-week-old BALB/c nude mice purchased from the Animal Resource Centre (Perth, Western Australia) were used in this study. Hepatocellular carcinoma was induced by intrahepatic implantation of 10^7^ Hepa1-6 cells into BALB/c nude mice via open surgical technique and imaging procedures were performed after 14 days [9]. Three mice per time point were randomly selected for time course studies. Each mouse was injected via the tail vein with 145.5 pmol/g of MSA-QDs (particle concentration was about 14.55 nmol/mL). The injection volume was 0.01 mL/g. Control mice were injected by the same volume of PBS. Serial sacrifices were carried out at 5 min, 1 h, 4 h, 8 h, 24 h, 7 days, and 30 days following dosing of MSA-QDs. Plasma samples were collected at each time point and were analyzed for quantification of QDs on the basis of Cd contents. Pharmacokinetic parameters were determined by compartmental analysis, and all the data were fitted by a two-compartment model.

### 2.3. Quantification of the QD Concentration in Plasma

QD concentration in mouse plasma was based on the determination of cadmium concentration by inductively coupled plasma-mass spectrometry (ICP-MS, Agilent 7700, Agilent Technologies, Tokyo, Japan). Briefly, plasma were collected, dried, weighted, and digested with 70% nitric acid on a hot plate at 100 °C. The Cd^2+^ concentration of these digested samples was measured using calibration curve ranged from 0 to 500 ng/mL [3].

### 2.4. MPM-FLIM

Short and long-term intravital images were collected from 30 s after injection using our published protocols [8,10]. MPM was performed using the DermaInspect system (Jen-Lab GmbH, Jena, Germany) equipped with an ultrashort (85 fs pulse width) pulsed mode-locked 80-MHz titanium sapphire laser (MaiTai, Spectra Physics, Mount View, CA, USA). The excitation wavelength was set to 740 nm for liver autofluorescence and 900 nm for QDs signals, with an emission signal range of 350 to 650 nm established through the use of a BG39 bandpass filter (BG39, Schott glass color filter, Schott MG, Mainz, Germany). Images were recorded with oil-immersion 40× objectives (Carl Zeiss, Germany). The laser power was set to 20 mW for 10× magnification imaging, and the acquisition time for obtaining the images was 7.4 s per frame. Each image was 179 × 179 μm wide at a resolution of 512 × 512 pixels. For mouse blood imaging, blood was collected after mouse scarification and was placed on the slide for imaging.

For fluorescence lifetime imaging (FLIM), a time-correlated single-photon counting (TCSPC) SPC-830 detector (Becker & Hickl, Berlin, Germany) was incorporated into the MPM system. The TCSPC module constructs a photon distribution across the x and y coordinates of the scan area. Each FLIM scan was performed using an exposure of 7.4 s at an acquisition image size of 214 × 214 μm. Fluorescence emission was spectrally resolved between linearly arranged photon counters through the use of dichroic filters in the beam path. The emission light was collected spectrally in a channel of 350 to 450 nm at the excitation of 740 nm for nicotinamide adenine dinucleotide (NADH), while the channel of 515 to 620 nm at the excitation of 740 nm for QD fluorescent signal.

### 2.5. Histological Analysis

Liver, kidney and spleen tissues were collected at day 7 or 30 after QDs intravenous injection in mice. These samples were fixed in formalin, then embedded in paraffin, sectioned, and stained with hematoxylin and eosin (H&E). Then the stained slides were examined by light microscopy.

### 2.6. Data Analysis

Quantitative analysis of the fluorescence intensity images was processed using ImageJ 1.44p (National Institutes of Health, Bethesda, MD, USA).

FLIM images were analyzed using SPCImage software 4.9.7 (Becker & Hickl, Berlin, Germany). Lifetime distributions for NADH were obtained by fitting photon count F(t) profiles from each image to a bi-exponential decay function (F(t) = α_1_e − t/τ_1_ + α_2_e − t/τ_2_). Two lifetimes, τ_1_ and τ_2_ represent the fast and slow decay lifetimes of free and protein-bound NADH, respectively. The amplitudes α_1_ and α_2_ represent the relative concentration fraction of nicotinamide adenine dinucleotide (NADH), where α_1_ + α_2_ = 1 [11]. τ_m_ is the weighted average lifetime calculated from τ_1_ and τ_2_ and their relative amplitudes (τ_m_ = α_1_τ_1_ + α_2_τ_2_).

## 3. Results

**Figure 1:**
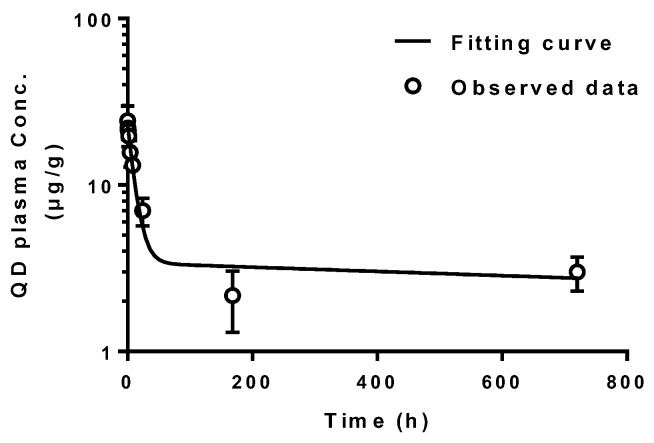
Plasma concentration of QDs following an intravenous injection detected by ICP-MS and it was fitted by a two-compartment model. Symbols represent the mean experimental data and error bars represent standard deviation. The solid lines are simulated by a two-compartment model.

These anionic ultrasmall QDs with the size of 3.5 nm in diameter that were mainly distributed in the blood vessels, and cannot extravasate, be taken up by parenchymal cells in organs or excreted into urine and feces up to 30 days [4]. Only a small amount of these particles were found to be taken up only by phagocytic cells (Kupffer cells and endothelial cells in liver, and mesangial cells in kidney) after injection [5,8]. In particular, our previous study has found that these QDs were gradually taken up by mesangial cells up to 30 days post-injection, which was detected by TEM imaging [5]. This result further supports QDs were not excreted through the body up to 30 days. We evaluated the QD concentrations in plasma by ICP-MS and they displayed a bi-exponential decay (Figure 1) in systemic blood circulation with long half-lives of 7.67 h (t_1/2α_) and 2363.19 h (t_1/2β_) (Table 1). Hence, these QDs circulated in the body for a long time and are suitable for the long-term intravital imaging of vasculature.

**Figure 2:**
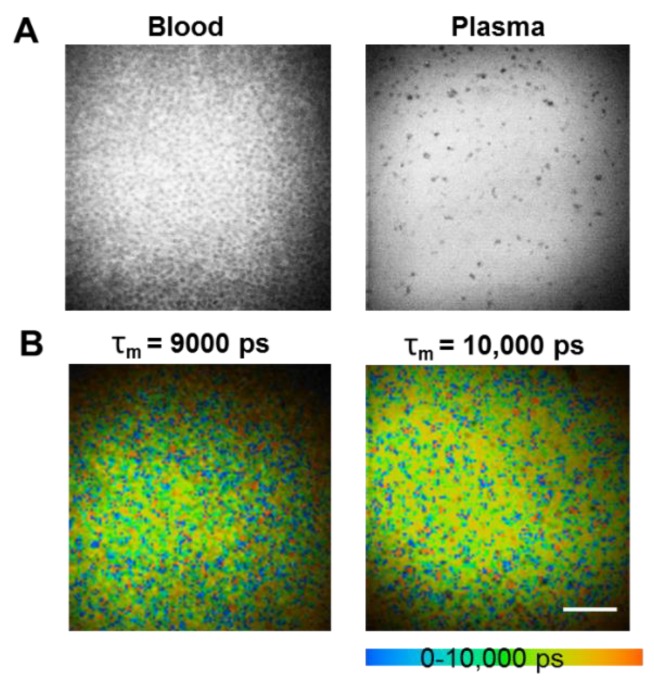
Fluorescence intensity image of mouse blood after QDs administration recorded at λ_Exc_/λ_Em_: 900/515 to 620 nm (**A**); Fluorescence lifetime properties of mouse blood after QDs administration measured by MPM-FLIM (**B**). Pseudocolored fluorescence lifetime image (τ_m_: 0–10,000 ps; blue-green-red) recorded at λ_Exc_/λ_Em_: 900/515 to 620 nm (scale bar: 160 μm).

We further performed in vitro experiments using MPM coupled with FLIM to determine the fluorescence lifetimes of mouse blood after QDs administration (Figure 2). The average weighted lifetime (τ_m_) was determined to be about 9 ns in mouse blood and 10 ns in plasma after QDs administration. These results indicated that the fluorescent propertis of these QDs were stable and they have longer lifetimes compared to normal tissue autofluorescence (2–3 ns) [3].

**Figure 3:**
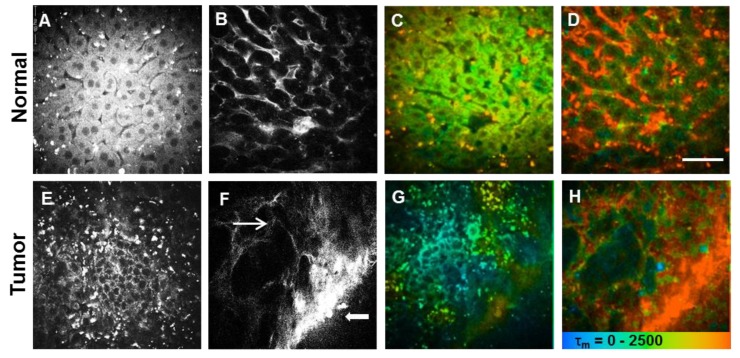
Multiphoton microscopy coupled with fluorescence lifetime imaging (MPM-FLIM) images of normal liver tissue and hepatocellular carcinoma after QDs injection. Narrow arrow indicates disordered and tortuous vasculature of hepatocellular carcinoma with inefficient blood perfusion and filled arrow indicates larger vessels connected to the tumor vasculature. (**A**,**E**) Fluorescence intensity image recorded at λ_Exc_/λ_Em_: 740/350 to 450 nm; (**B**,**F**) Fluorescence intensity image recorded at λ_Exc_/λ_Em_: 900/515 to 620 nm; (**C**,**G**) Pseudocolored fluorescence lifetime image (τ_m_: 0–2500 ps; blue-green-red) recorded at λ_Exc_/λ_Em_: 740/350 to 450 nm or (**D**,**H**) recorded at λ_Exc_/λ_Em_: 900/515 to 620 nm. (Scale bar: 40 μm).

**Figure 4:**
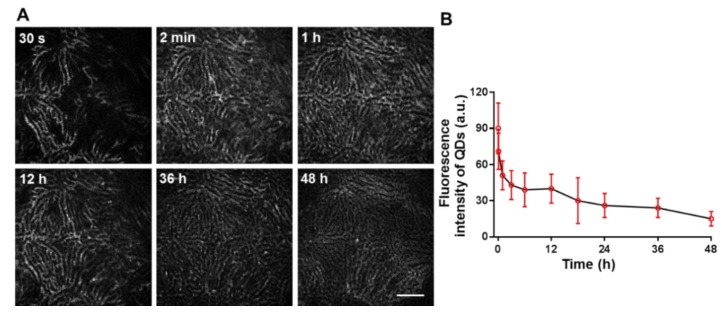
Time profile of QDs intensity in vasculature of normal liver after bolus injection. (**A**) Real-time hepatic disposition of QDs, (Scale bar: 160 μm); (**B**) Time profile of QDs intensity per pixel. The symbols represent measured data and the line represents the connecting curve. Error bar represents the standard deviation (*n* = 3).

Liver images were obtained at the excitation wavelength of 900 nm to selectively capture QD fluorescent signal since liver autofluorescence of NADH is absent at this excitation wavelength [3]. As shown in Figure 3B,F, vasculatures of normal liver tissue and hepatocellular carcinoma were clearly visualized by highly intense fluorescence signals of QDs after 30 min of intravenous injection. Unlike highly regulated and controlled sinusoids in normal liver tissues, tumor tissues exhibited disordered and tortuous vasculature, mainly due to tumor aniogenesis and lack of blood perfusion. Neovessels were found to be connected with larger vessels. These images further confirmed that QDs were retained in the vasculature and were not taken up by hepatocytes or tumor cells (Figure 3A,B,E,F). As shown in Figure 4, the fluorescence of QDs reached maximum at 2 min post-injection, and was evenly distributed in blood vessels. The QDs labeled vascular images were obvious up to 36 h after injection, but QD signals gradually disappeared at 48 h post-injection.

In addition to fluorescent signals, FLIM could map the spatial distribution of fluorophores based on fluorescence lifetime, the average time of an electron stays in the excited state before returning to the ground state [11]. Lifetime changes also can be used to differentiate fluorophores from biological environments and also detect environmental changes, such as protein binding or pH [10]. These anionic ultrasmall QDs are particularly suitable for FLIM because their fluorescence lifetime (typically ≥10 ns) is much longer than those of organ autofluorescence (2–3 ns) [3], which also have been confirmed in Figure 2. As shown in Figure 3D,H, FLIM images further clearly confirmed the QDs labeled vasculatures of normal liver tissue and hepatocellular carcinoma, where orange color represents longer lifetime of QDs (Figure 3D,H) and green color (Figure 3C,G) represents shorter lifetimes of autofluorescence from hepatocytes or tumor cells.

**Figure 5:**
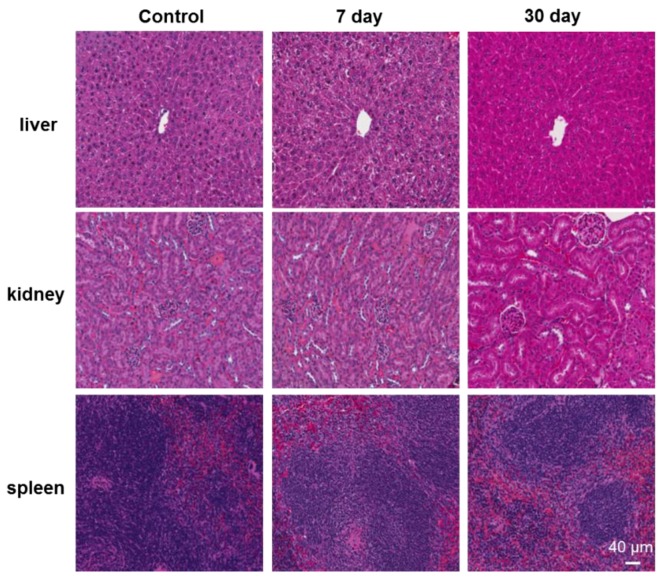
Representative organ histology images of control and QDs treated mice. QDs treated organs were collected 7 days or 30 days after intravenous injection of QDs. (Scale bar: 40 μm).

Finally, we did the histological analysis of key organs of mice treated by QDs, which allows for detailed microscopic evaluation and histological assessment of tissue interactions. Since QDs are mainly accumulated in the liver, spleen, and kidney for a long period, it is necessary to understand their toxicity in these organs. As shown in Figure 5, there were no apparent histopathological abnormalities or lesions observed in these organs exposed with QDs. Hepatocytes were observed to be normal and there were no signs of inflammatory response in QDs treated tissues. The glomerular structure of the kidney was clear and no pathological changes were observed in the spleen. H&E staining images of such organs showed the same properties as those of control tissues, with no signs of overt degeneration, inflammation and necrosis in any of examined tissues. The lower toxicity of these QDs to the organs could be attributed to the formation of CdS shell on the core CdTe of QDs [12].

## 4. Discussion

In this study, we found that the anionic ultrasmall QDs present new opportunities for intravital vascular imaging as they retain in vascular for a long time after systematic injection without uptake by parenchymal cells. Compared to conventional in vivo imaging, MPM has the advantages of less photobleaching and photodamage, and has considerably enhanced imaging penetration depth due to less scattered multiphoton excitation in samples [10]. Fresh, unprocessed and unstained organs can be imaged in vivo or ex vivo by MPM [13]. Most vascular contrast agents are transient imaging reagents with imaging time windows ranging from a few minutes to several hours, depending on the properties of the probe. In contrast, ultrasmall inorganic NPs have longer half-lives in the body in comparison with larger NPs, organic fluorescent dyes or bovine serum albumin (BSA) and transferrin conjugated vasculature agents [4]. We have previously reported that cationic QDs of similar size can be readily excreted into urine, while anionic ultrasmall QDs have prolonged blood circulation and high photostability [3], which allows repeated and enhanced angiogenesis imaging. Compared to conventional technique for quantifying blood flow, MPM has been opening up a new avenue for simultaneous imaging depiction of the cellular and extracellular microstructures in tissues [14]. Here, we reported the pharmacokinetics of these QDs, and demonstrated that their fluorescence remained in vasculature up to 36 h after injection under MPM, allowing long-term intravital imaging. These QDs have advantages over organic nanoparticles (e.g., polymers and liposomes) for tumor angiogenesis imaging since they have less extravasation in tumor due to the rigid cores. However, most of these published or commercialized QDs will readily leak from the capillaries within 6 h [15]. It is of interest to note that our non-targeted QDs show little or no extravasation from the vasculature up to 36 h post introduction into the tumor xenograft bearing mice. These NPs can be used separately, or in combination with other proteins/antibodies to the in vivo imaging system.

Vascular imaging is of particular importance in tumor biology where imaging can be used to study the growth and progression of cancer, and assess the efficacy of anti-tumor and anti-angiogenesis drugs [16]. Once the diameter of a tumor reaches 1 to 2 mm, diffusion of nutrients and oxygen from surrounding host vasculature becomes insufficient, leading to angiogenesis, the formation of new blood vessels from preexisting vasculature [17]. Tumor-induced angiogenesis is connected to the host’s circulatory system, and plays a key role in cancer invasion and metastasis. Therefore, vasculature of cancer could be used as diagnostic and prognostic markers and the excellent targets for chemotherapy [17]. Since the angiogenesis of tumors highly reached the surface [18], imaging of the vasculature near the tumor surface is feasible for detecting angiogenesis using MPM-FLIM [19].

In summary, we show that anionic ultrasmall QDs can be used to visualize the in vivo vasculature in normal and cancerous livers in mice, with cellular resolution (~1 µm), up to 36 h after intravenous injection. MPM coupled with FLIM has great potential as emerging tools in diagnosis and monitoring of treatment response in liver cancer.

## Figures and Tables

**Table 1:** Pharmacokinetic parameters of quantum dots (QDs) after intravenous injection fitted by a two-compartment model.

Parameter (Unit)	Value	Parameter (Unit)	Value
CL (mL/h)	0.006	k_12_ (h^−1^)	0.075
Vc (mL)	3.11	k_21_ (h^−1^)	0.014
CLd (mL/h)	0.23	t_1/2α_ (h)	7.67
Vss (mL)	20.38	t_1/2β_ (h)	2363.19

## References

[1] Lewis J.D., Destito G., Zijlstra A., Gonzalez M.J., Quigley J.P., Manchester M., Stuhlmann H. (2006). Viral nanoparticles as tools for intravital vascular imaging. Nat. Med..

[2] Hilderbrand S.A., Weissleder R. (2010). Near-infrared fluorescence: Application to in vivo molecular imaging. Curr. Opin. Chem. Biol..

[3] Liang X., Grice J.E., Zhu Y., Liu D., Sanchez W.Y., Li Z., Crawford D.H., Le Couteur D.G., Cogger V.C., Liu X. (2014). Intravital Multiphoton Imaging of the Selective Uptake of Water-Dispersible Quantum Dots into Sinusoidal Liver Cells. Small.

[4] Liang X.W., Wang H.L., Grice J.E., Li L., Liu X., Xu Z.P., Roberts M.S. (2016). Physiologically Based Pharmacokinetic Model for Long-Circulating Inorganic Nanoparticles. Nano Lett..

[5] Liang X.W., Wang H.L., Zhu Y., Zhang R., Cogger V.C., Liu X., Xu Z.P., Grice J.E., Roberts M.S. (2016). Short- and Long-Term Tracking of Anionic Ultrasmall Nanoparticles in Kidney. Acs Nano.

[6] Cai W.B., Shin D.W., Chen K., Gheysens O., Cao Q.Z., Wang S.X., Gambhir S.S., Chen X.Y. (2006). Peptide-labeled near-infrared quantum dots for imaging tumor vasculature in living subjects. Nano Lett..

[7] Smith B.R., Cheng Z., De A., Koh A.L., Sinclair R., Gambhir S.S. (2008). Real-time intravital imaging of RGD-quantum dot binding to luminal endothelium in mouse tumor neovasculature. Nano Lett..

[8] Wang H.L., Liang X.W., Mohammed Y.H., Thomas J.A., Bridle K.R., Thorling C.A., Grice J.E., Xu Z.P., Liu X., Crawford D.H.G. (2015). Real-time histology in liver disease using multiphoton microscopy with fluorescence lifetime imaging. Biomed. Opt. Express.

[9] Wang H., Thorling C.A., Xu Z.P., Crawford D.H.G., Liang X., Liu X., Roberts M.S. (2017). Visualization and Modeling of the In Vivo Distribution of Mesenchymal Stem Cells. Curr. Protoc. Stem Cell Biol..

[10] Thorling C.A., Liu X., Burczynski F.J., Fletcher L.M., Gobe G.C., Roberts M.S. (2011). Multiphoton microscopy can visualize zonal damage and decreased cellular metabolic activity in hepatic ischemia-reperfusion injury in rats. J. Biomed. Opt..

[11] Lakowicz J.R., Szmacinski H., Nowaczyk K., Berndt K.W., Johnson M. (1992). Fluorescence Lifetime Imaging. Anal. Biochem..

[12] Zhu Y., Li Z., Chen M., Cooper H.M., Lu G.Q., Xu Z.P. (2013). One-pot preparation of highly fluorescent cadmium telluride/cadmium sulfide quantum dots under neutral-pH condition for biological applications. J. Colloid Interf. Sci..

[13] Zipfel W.R., Williams R.M., Webb W.W. (2003). Nonlinear magic: multiphoton microscopy in the biosciences. Nat. Biotechnol..

[14] Wang B.G., Konig K., Halbhuber K.J. (2010). Two-photon microscopy of deep intravital tissues and its merits in clinical research. J. Microsc-Oxford.

[15] Kairdolf B.A., Smith A.M., Stokes T.H., Wang M.D., Young A.N., Nie S. (2013). Semiconductor quantum dots for bioimaging and biodiagnostic applications. Annu. Rev. Anal. Chem. (Palo Alto Calif.).

[16] Bergers G., Benjamin L.E. (2003). Tumorigenesis and the angiogenic switch. Nat. Rev. Cancer.

[17] Liu Z.A., Peng R. (2010). Inorganic nanomaterials for tumor angiogenesis imaging. Eur. J. Nucl. Med. Mol. I.

[18] Cai W.B., Chen X.Y. (2008). Multimodality molecular imaging of tumor angiogenesis. J. Nucl. Med..

[19] Jain R.K., Munn L.L., Fukumura D. (2002). Dissecting tumour pathophysiology using intravital microscopy. Nat. Rev. Cancer.

